# Supplementary material for: Bayesian refinement of protein structures and ensembles against SAXS data using molecular dynamics
Source: PLoS Comput Biol. 2017 Oct 18;13(10):e1005800. doi: 10.1371/journal.pcbi.1005800 (PMC5662244; doi:10.1371/journal.pcbi.1005800)
Supplement: S1 Archive — (BZ2) [file pcbi.1005800.s011.tar.bz2 › gromacs_4.62_bayesian_saxs_refi/share/html/online/g_bundle.html]

g\_bundle


| g\_bundle Main Table of Contents |

**VERSION 4.6  
Sat 19 Jan 2013**

---

### Description

g\_bundle analyzes bundles of axes. The axes can be for instance
helix axes. The program reads two index groups and divides both
of them in -na parts. The centers of mass of these parts
define the tops and bottoms of the axes.
Several quantities are written to file:
the axis length, the distance and the z-shift of the axis mid-points
with respect to the average center of all axes, the total tilt,
the radial tilt and the lateral tilt with respect to the average axis.

With options -ok, -okr and -okl the total,
radial and lateral kinks of the axes are plotted. An extra index
group of kink atoms is required, which is also divided into -na
parts. The kink angle is defined as the angle between the kink-top and
the bottom-kink vectors.

With option -oa the top, mid (or kink when -ok is set)
and bottom points of each axis
are written to a .pdb file each frame. The residue numbers correspond
to the axis numbers. When viewing this file with Rasmol, use the
command line option -nmrpdb, and type set axis true to
display the reference axis.

### Files

| option | filename | type | description |
| --- | --- | --- | --- |
| **-f** | traj.xtc | Input | Trajectory: xtc trr trj gro g96 pdb cpt |
| **-s** | topol.tpr | Input | Structure+mass(db): tpr tpb tpa gro g96 pdb |
| **-n** | index.ndx | Input, Opt. | Index file |
| **-ol** | bun\_len.xvg | Output | xvgr/xmgr file |
| **-od** | bun\_dist.xvg | Output | xvgr/xmgr file |
| **-oz** | bun\_z.xvg | Output | xvgr/xmgr file |
| **-ot** | bun\_tilt.xvg | Output | xvgr/xmgr file |
| **-otr** | bun\_tiltr.xvg | Output | xvgr/xmgr file |
| **-otl** | bun\_tiltl.xvg | Output | xvgr/xmgr file |
| **-ok** | bun\_kink.xvg | Output, Opt. | xvgr/xmgr file |
| **-okr** | bun\_kinkr.xvg | Output, Opt. | xvgr/xmgr file |
| **-okl** | bun\_kinkl.xvg | Output, Opt. | xvgr/xmgr file |
| **-oa** | axes.pdb | Output, Opt. | Protein data bank file |

### Other options

| option | type | default | description |
| --- | --- | --- | --- |
| **-[no]h** | bool | no | Print help info and quit || **-[no]version** | bool | no | Print version info and quit || **-nice** | int | 19 | Set the nicelevel || **-b** | time | 0 | First frame (ps) to read from trajectory || **-e** | time | 0 | Last frame (ps) to read from trajectory || **-dt** | time | 0 | Only use frame when t MOD dt = first time (ps) || **-tu** | enum | ps | Time unit: fs, ps, ns, us, ms or s || **-xvg** | enum | xmgrace | xvg plot formatting: xmgrace, xmgr or none || **-na** | int | 0 | Number of axes || **-[no]z** | bool | no | Use the z-axis as reference instead of the average axis |

---

http://www.gromacs.org  
gromacs@gromacs.org
